# Supplementary material for: A Waterborne, Flexible, and Highly Conductive Silver Ink for Ultra-Rapid Fabrication of Epidermal Electronics
Source: Sensors (Basel). 2025 Mar 27;25(7):2092. doi: 10.3390/s25072092 (PMC11991362; doi:10.3390/s25072092)
Supplement: Supplementary file 1 [file sensors-25-02092-s001.zip › sensors-3522720-supplementary.pdf]

-Supplementary File-

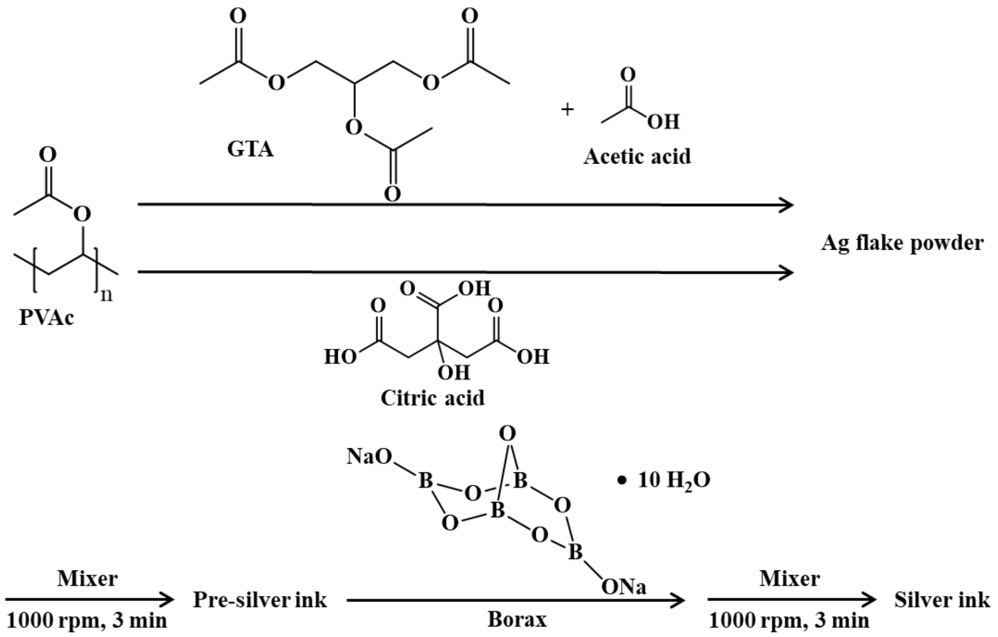

Scheme S1. The chemical structures of the materials and the fabrication of the silver ink.

Table S1. Composition of each sample.

Sample	Glue-All (g)	GTA (g)	Acetic acid (g)	Citric acid (g)	Borax (g)	5 % Borax$_{(aq)}$ (g)	Ag flake powder (g)
**Glue-All**		--	--	--	--		--
**GTA**		0.68	--	--	--		--
**GTA+Ag**		0.68	--	--	--		10.99
**CA**		--	--	1.548	--		--
**CA+Ag**		--	--	1.548	--		10.13
**Borax1**	15	0.68	0.032	--	0.068	1.377	13.65
**Borax2**		1.54	0.036	--	0.077	1.548	13.12
**Borax3**		2.65	0.042	--	0.088	1.77	12.43
**Borax4**		--	--	0.688	0.068	1.377	13.58
**Borax5**		--	--	1.548	0.077	1.548	13.04
**Borax6**		--	--	2.655	0.088	1.77	12.35

Table S2. The comparison table of different conductive ink

Paper	Filler	Resistivity (ohms*m)
MWCNTs-GNPs Reinforced TPU Composites with Thermal and Electrical Conductivity: Low-Temperature Controlled DIW Forming	Graphene + Carbon Nanotubes	18.125
Piezo-resistive Sensor of Auxetic Structures Based on 3D-Printed TPU Coated by Castor-Oil-Based Waterborne	Graphene	3.86
The Influence of the Matrix Selection and the Unification Process on the Key Parameters of the Conductive Graphene layers on a Flexible substrate	Graphene	2.6
Efficient Fabrication of Highly stretchable and Ultrasensitive Thermoplastic Polyurethane/Carbon Nanotube Foam with Anisotropic Pore Structure for Human Motion Monitoring	Carbon Nanotube	10
3D Printing of Carbon Nanotube (CNT)/Thermoplastic Polyurethane (TPU) Functional Composites and Preparation of Highly Sensitive, Wide-range Detectable, and Flexible Capacitive Sensor Dielectric Layers via Fused Deposition Modeling (FDM)	Carbon Nanotube	72
Polyaniline/thermoplastic polyurethane blends: Preparation and evaluation of electrical conductivity	Polyaniline	0.1
The role of lithium salts in the conductivity and phase morphology of a thermoplastic polyurethane	Lithium Salt	10000
Morphology and ionic conductivity of thermoplastic polyurethane electrolytes.	Lithium Salt	10
High-Performance Stretchable Conductive Composite Fibers from Surface-Modified Silver Nanowires and Thermoplastic Polyurethane by Wet Spinning	Ag Nanowire	0.000001
Electrically Conductive Adhesive Based on Thermoplastic Polyurethane Filled with Silver Flakes and Carbon Nanotubes	Ag Flakes	0.000004
Our Work	Ag Flakes	0.0000009

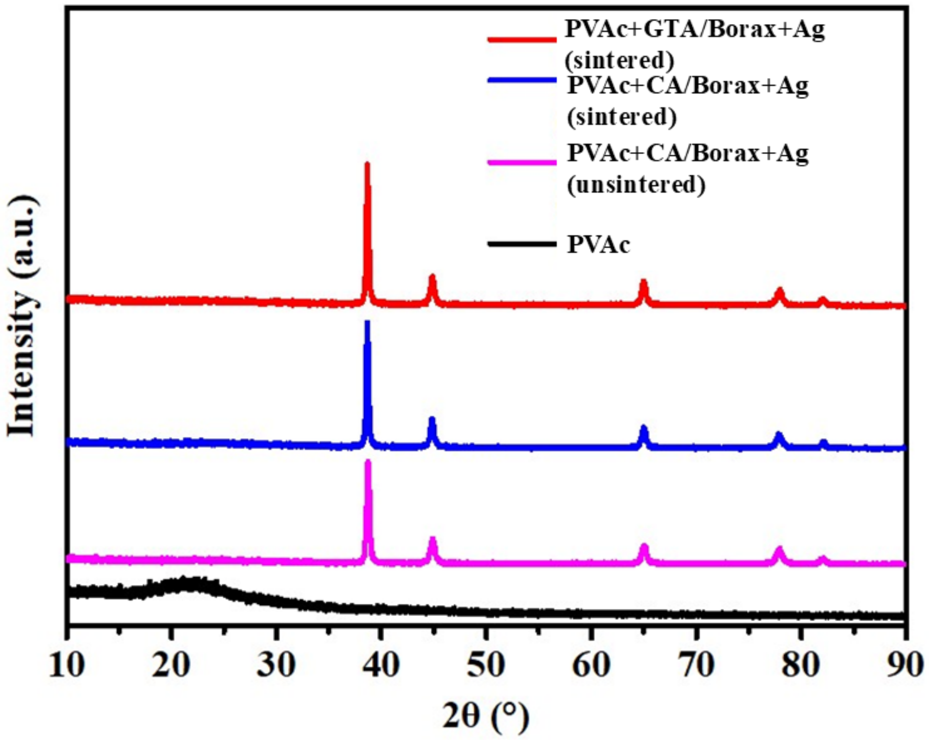

Figure S1. XRD patterns of the various inks that were sintered at 100 °C for 30 min and the comercial Ag flakes.

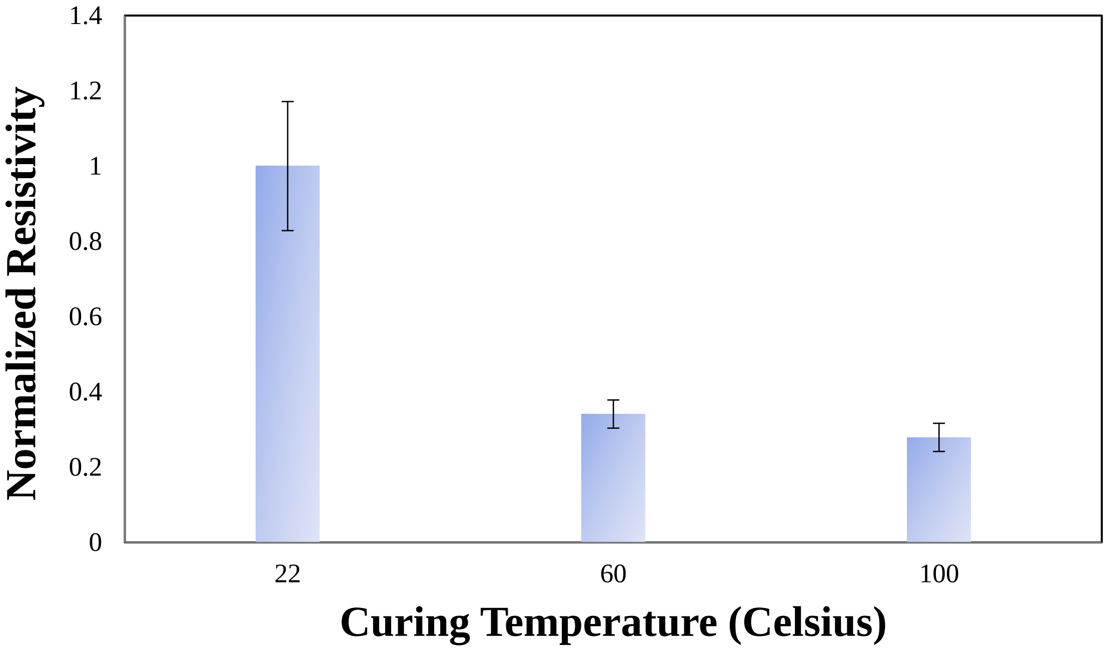

Figure S2. Resistivity in different curing temperature.

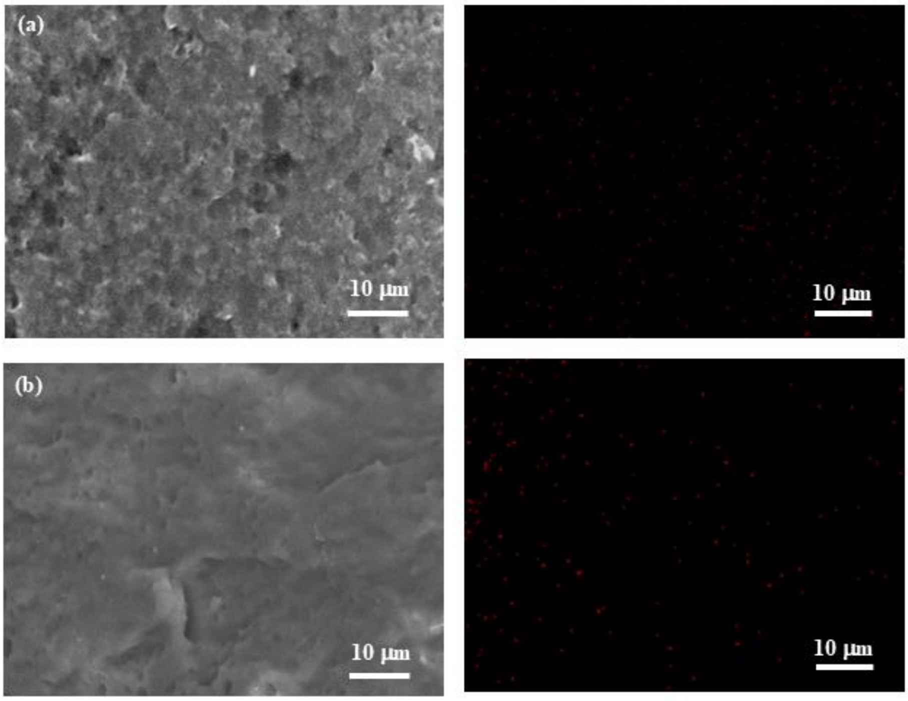

Figure S3. SEM and EDS mapping of group Glue All and GTA

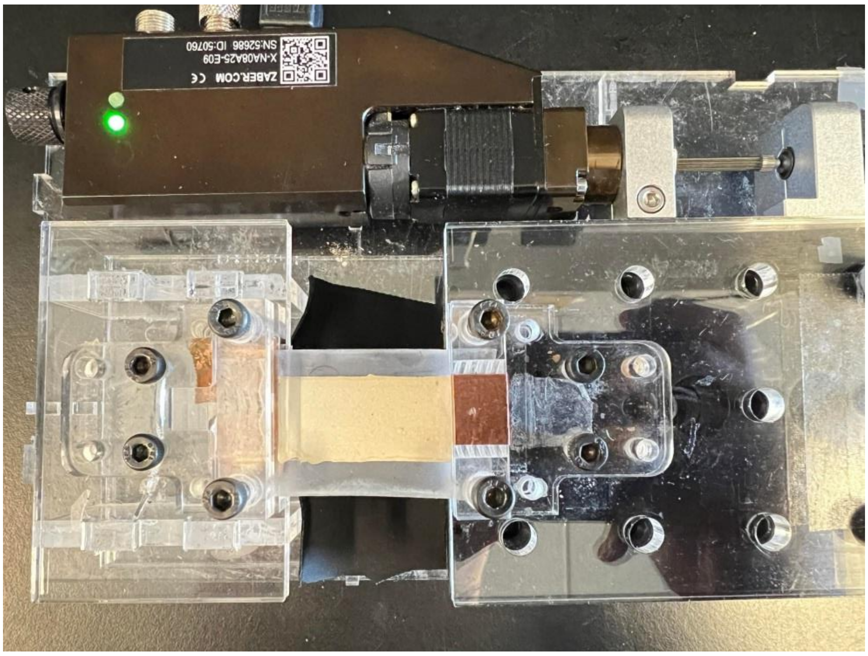

Figure S4. The elongation setup on TPU substrate to test the strain to failure test

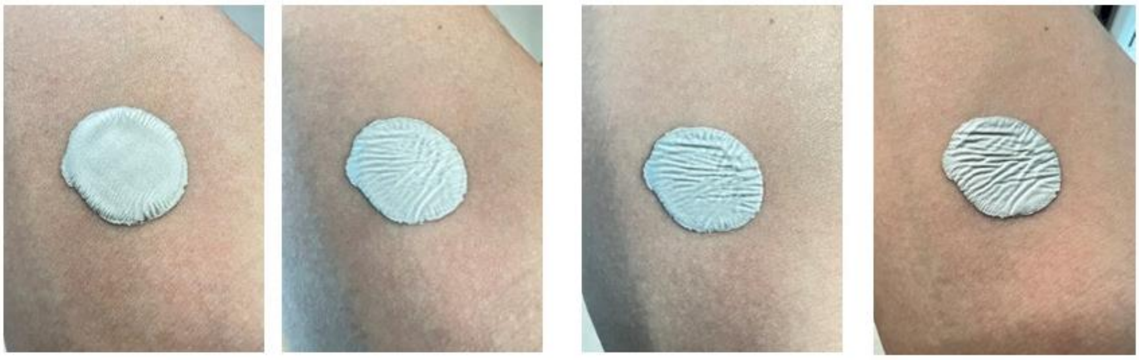

Figure S5. Paint on stability characteristic under different curing time

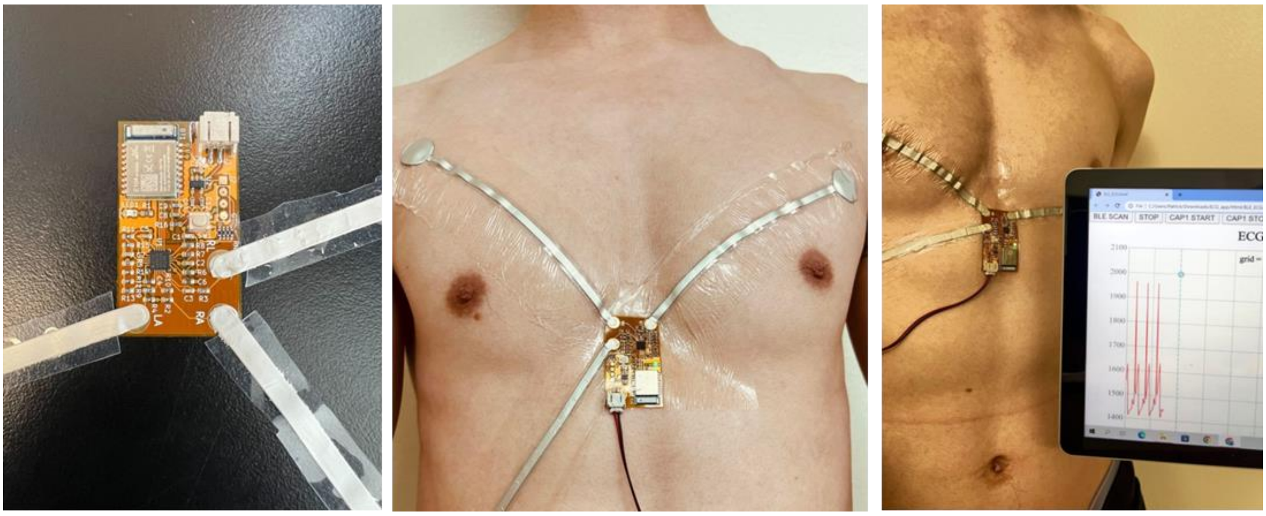

Figure S6. Wireless ECG setup

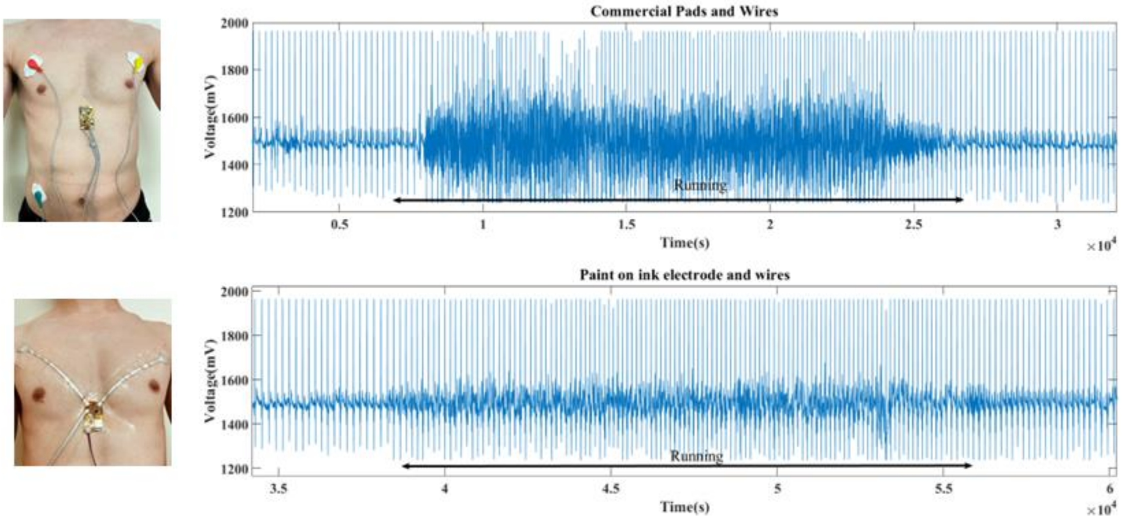

Figure S7. Raw data of ECG during running

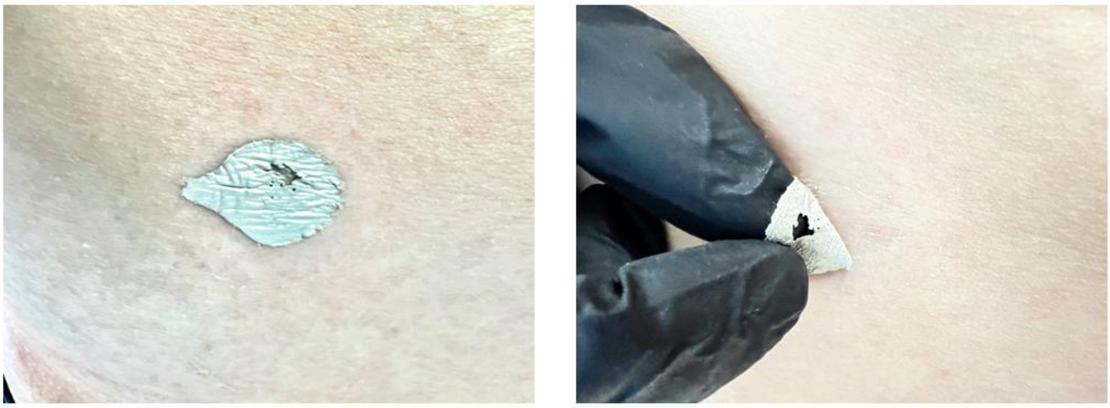

Figure S8. No irritation after putting the ink electrode on skin for an hour

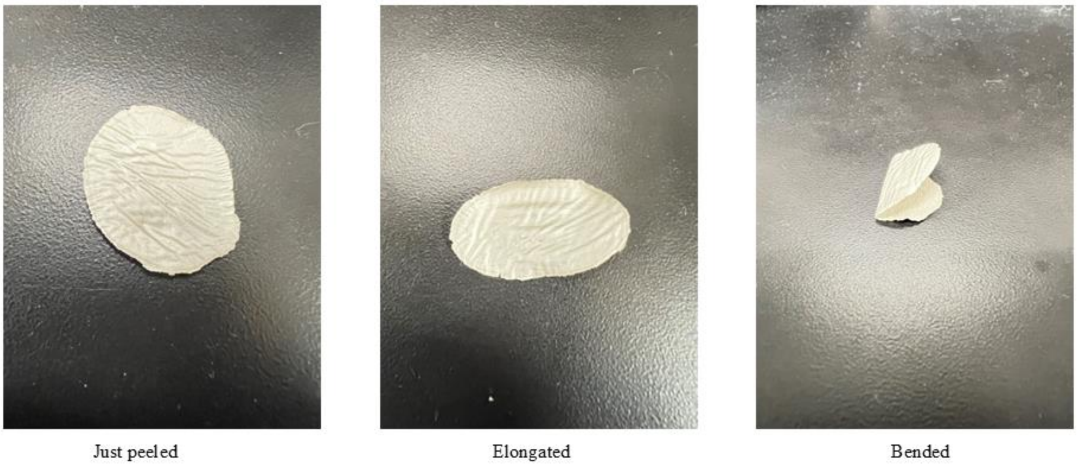

Figure S9. Electrodes after 90 minutes
